# Synchronous isolated gastric metastases from ascending colon carcinoma: A case report

**DOI:** 10.1097/MD.0000000000032476

**Published:** 2022-12-23

**Authors:** Bin Yang, Zhonghua Gan, Shulan Liu, Guangyan Si

**Affiliations:** a The Affiliated Traditional Chinese Medicine Hospital of Southwest Medical University, 182 chunhui RD, Lu Zhou, Sichuan, People’s Republic of China.

**Keywords:** case report, colon carcinoma, gastric metastases, isolated, synchronous

## Abstract

Gastric metastases (GMs) are rare and often accompanied with synchronous metastases of other organs. Synchronous isolated GMs from ascending colon carcinoma are uncommon and rarely studied. GMs may be confused with primary gastric tumors.

**Methods:**

A 45-year-old man presented to our hospital with abdominal distension

and anal pendant expansion. The abdominal physical examination was negative. The positive fecal occult blood test and the negative tumor marker were obtained. Colonoscopy and gastroduodenoscopy revealed a polypoidal lesion in the ascending colon and a polypoid mass in the gastric body, respectively. CT showed the thickened wall of ascending colon and polypoid mass in the gastric body with homogenous enhancement. Additionally, synchronous gastric metastases from the ascending colon carcinoma were confirmed by pathology after laparoscopic right hemicolectomy and partial gastrectomy. After 13 individual doses of fluorouracil (2.8 g/time), calcium leucovorin (0.8 g/time), and oxaliplatin (85 mg/time), the patient was discharged without any discomfort, without any additional metastases detected during the following 18 months.1.

**Results:**

A rare case of synchronous isolated gastric metastasis from ascending colon carcinoma was confirmed by computed tomography (CT) and pathological diagnosis.

**Conclusion:**

GM may appear as a polypoid lesion. Surgery combined with chemotherapy may improve the prognosis in patients with synchronous isolated GM.

## 1. Introduction

The reported incidence of gastric metastasis (GM) is only around 0.3% (9/2579).^[1]^ The primary tumor of GM usually originates from breast cancer, lung cancer, renal cell carcinoma, malignant melanoma^[2]^ and rarely from reproductive system cancers, hepatocellular carcinoma, Merkel cell carcinoma, anaplastic thyroid carcinoma, colon cancer, etc.^[3–7]^ Therefore, GM is often accompanied with metastasis of other organs, such as the lung, liver, brain, and bone. However, synchronous isolated GM from ascending colon carcinoma is uncommon, with only a few cases reported worldwide.^[8]^ It should be noted that GM may be misdiagnosed as other gastric tumors because it has not been well understood. We report a synchronous GM case from ascending colon carcinoma confirmed by pathology. To our knowledge, a polypoid lesion of the stomach is a manifestation of GM.

We present the following case in accordance with the CARE reporting checklist.

## 2. Case presentation

### 2.1. Chief complaints

A 45-year-old man presented to our hospital with a 6-month history of abdominal distension and anal pendant expansion.

### 2.2. History of present illness

Paroxysmal abdominal distension and pain were accompanied by a sense of anal pendant expansion, and the pain was relieved after using the toilet. Paroxysmal abdominal distension and pain developed progressively in less than half a month. The patient demonstrated no nausea, vomiting, cold or chills, and stool was neither mucoid nor purulent.

### 2.3. History of past illness

The patient had no history of hypertension, diabetes, coronary heart disease, tuberculosis, or hepatitis.

### 2.4. Personal and family history

The patient had no history of smoking or drinking, with a negative family medical history of hereditary diseases.

### 2.5. Physical examination

The abdomen was soft and flat, with no pain or tenderness, whereas the liver and spleen were not palpable.

### 2.6. Laboratory examinations

A positive fecal occult blood test was obtained. However, the levels of the tumor markers, CA19-9, CA153, CA-125, and carcinoembryonic antigen, were normal. Blood tests for liver and kidney function and electrolyte levels were negative. Ultrasonic examination showed that the liver, gallbladder, and spleen were unremarkable.

### 2.7. Gastrointestinal endoscopy

**Figure 1.**
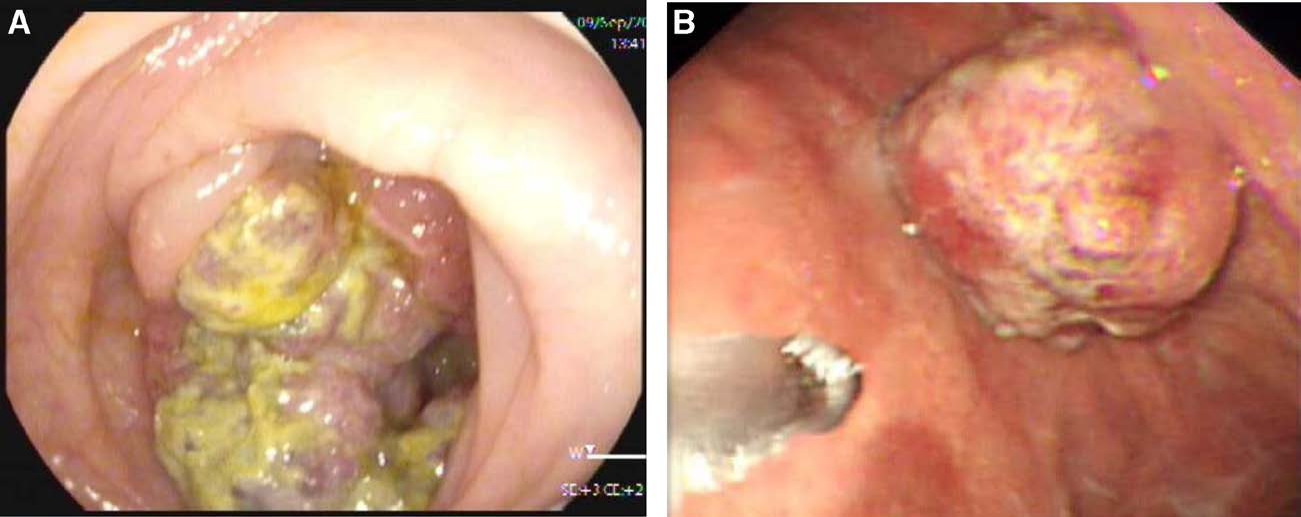
Colonoscopy and gastroduodenoscopy images from the case of a 45-year-old man with synchronous isolated gastric metastasis. (A) A polypoidal lesion in the ascending colon, occupying two-thirds of colon wall. (B) A 1.5 × 2.0 cm polypoid mass in the greater curvature of the gastric body.

Colonoscopy revealed a polypoidal lesion in the ascending colon with a hard texture and high tendency to bleed (Fig. 1a). This mass occupied two-thirds of the colon wall. Gastroduodenoscopy revealed a 1.5 × 2.0 cm polypoid mass in the greater curvature of the gastric body (Fig. 1b).

### 2.8. Imaging examinations

**Figure 2.**
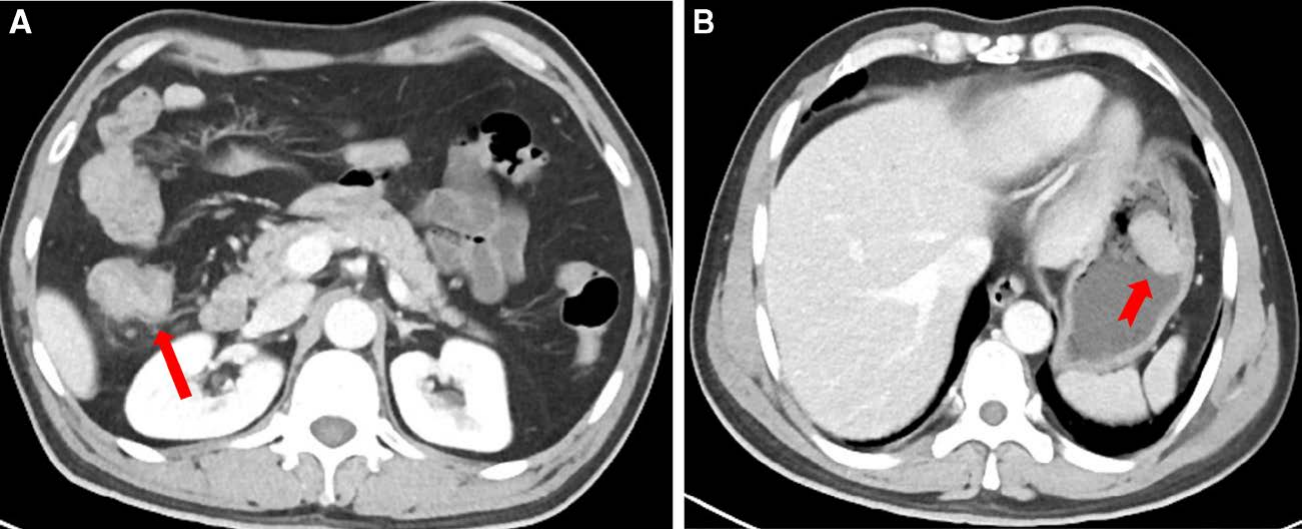
Computed tomography images from the case of a 45-year-old man with synchronous isolated gastric metastasis. (A) Contrast-enhanced CT image shows the thickened wall (red arrow) in the ascending colon persistent significant enhancement in the portal venous phase. (B) Contrast-enhanced CT image shows a polypoid mass (red dovetail arrow) with persistent enhancement in the portal venous phase.

Computed tomography (CT) was performed during hospitalization. The results showed an asymmetrically thickened wall in the ascending colon (Fig. 2a) and a 2.1 × 3.5 cm polypoid mass in the greater curvature of the gastric body (Fig. 2b). All the lesions were homogeneously isodense on nonenhanced CT images and homogeneously enhanced (Fig. 2a and b). Some small lymph nodes were seen around the ascending colon, but none were observed in the vicinity of the stomach. There were no intra-abdominal fluid collections or distant metastasis in the thorax and abdomen.

## 3. Treatment

Laparoscopic right hemicolectomy and partial gastrectomy were performed. During hospitalization, the patient received treatments for infection prevention (amikacin sulphate) and liver protection (reduced glutathione and compound glycyrrhizin). After surgery, the patient recovered well and was discharged from the hospital. After 13 individual doses of fluorouracil (2.8 g/time), calcium leucovorin (0.8 g/time), and oxaliplatin (85 mg/time), the patient underwent CT, which was negative. The whole-body CT conducted 18 months after the treatment revealed no recurrence or distance metastasis.

### 3.1. Pathological findings

**Figure 3.**
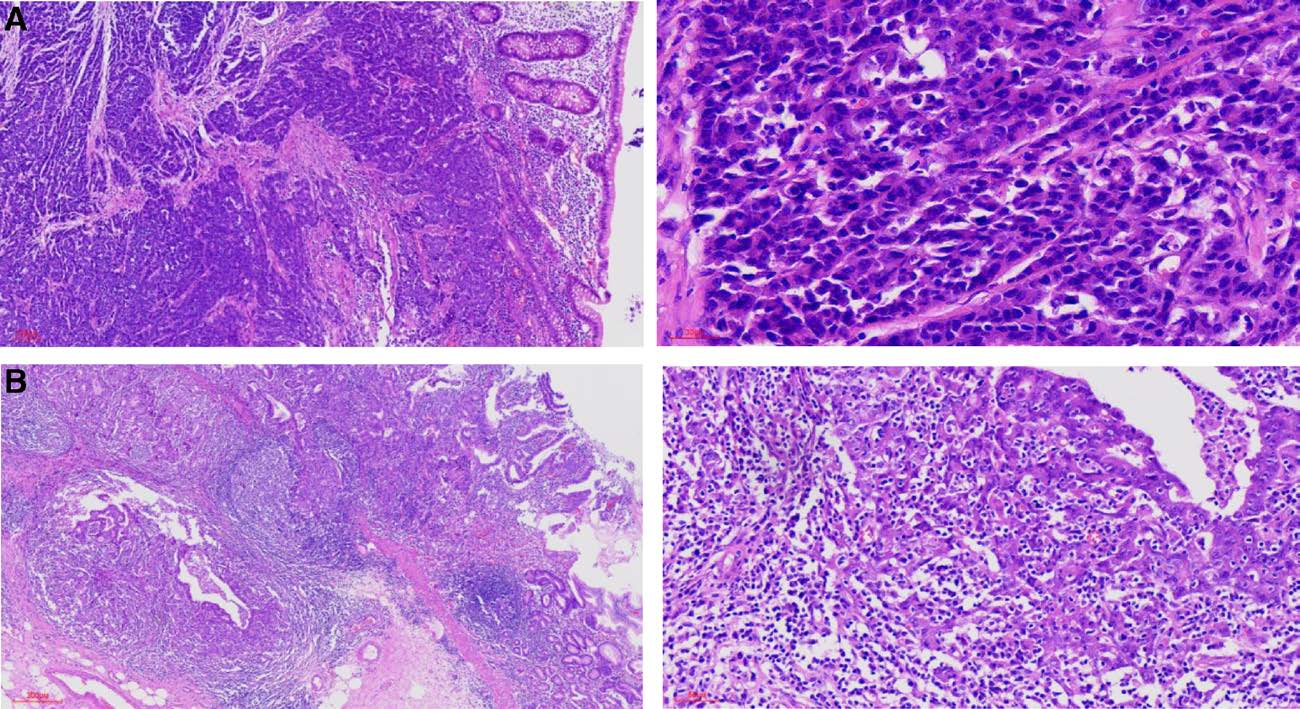
Images of Histological and immunohistochemical staining of ascending colon cancer from the case of a 45-year-old man with synchronous isolated gastric metastasis. (A) Hematoxylin and eosin staining (×100 and × 400) showing adenocarcinoma tumor cells (nos, G3) from the ascending colon. The tumor cells arranged in a cord pattern, and were distributed diffusely with rare gland duct differentiation. The nuclear/cytosolic ratio was high. Deep staining and the nuclear division were observed in high numbers. (B) Hematoxylin and eosin staining (×100 and × 400) showing adenocarcinoma tumor cells of the stomach. The cytologic atypia of tumor cells was obvious, and the tumors had a small amount of gland duct differentiation zone.

Ascending colon carcinoma (Fig. 3a) with synchronous GM (Fig. 3b) was confirmed by pathology. Ascending colon carcinoma had invaded the deep muscular layer with intravascular cancer thrombus, without lymph node metastasis and nerve invasion. Immunohistochemical examination of ascending colon carcinoma revealed negative for CK7, Vimentin, Melan-A, S-100, AFP, CgA and Syn, weakly positive for Villin, and positive for P53 (10% of cells positive), CDX-2, and CK20.

## 4. Discussion and conclusion

Malignant tumors, such as breast cancer, lung cancer, renal cell carcinoma, malignant melanoma, can metastasize to the stomach.^[2]^ However, metastasis to the stomach is uncommon, especially that originates from colon cancers. According to the report of Terashima et al,^[8]^ there were 14 GM cases originating from colon cancer in 2019 in the Fukushima Journal of Medical Science.

The clinical symptoms of GM are nonspecific,^[9]^ including dyspepsia, anorexia, abdominal distension, black stool, nausea, vomiting, upper abdominal pain, early satiety, and bleeding, and even gastric perforation.^[10–12]^ However, the most common symptom is abdominal pain. Our patient presented with a 6-month history of abdominal distension and anal pendant expansion, which may be caused by colon cancer.

GM can occur anywhere in the stomach, but mainly in the upper two-thirds of the stomach. They can be single or multiple and synchronous or metachronous, while they are mainly single and metachronous.^[10]^ Our patient had single synchronous stomach metastasis.

There are 4 types of GM.^[13]^ The first type comprises polypoid lesions, a nodule or mass, which is easily confused with gastric polyps. The second type is ulcerative mass, which is the typical imaging feature of GM. This mass is mainly in the submucosa of the stomach, manifesting as local gastric wall thickening with ulceration at the top of the lesion and normal adjacent or surface mucosa. This is described as a “bull’s-eye sign” or “crateriform ulcer” on gastrointestinal endoscopy.^[14]^ It is easily confused with gastric stromal tumors. The third type involves a submucosal nodule in the gastric wall.^[15]^ It is easily confused with gastric stromal tumors, neurinoma, and liomyoma. The final type is diffuse gastric wall thickening, also called “linitis plastica,”^[16]^ which is prone to be confused with diffuse gastric cancer. Except for the diffuse gastric wall thickening type, the mucosa and adjacent gastric wall of the other GM types usually appear normal, without enlarged lymph nodes around the stomach. GM may occur alone or in association with other metastatic sites such as the lung, liver, brain, and adrenal glands, and the latter is more common. The current case belongs to the first type and has a polypoid mass with homogeneous enhancement.

Metastasis to the gastric wall occurs in several ways, such as direct invasion, hematogenous metastasis, lymphatic metastasis, and intraoperative implantation.^[17]^ Among them, hematogenous and lymphatic metastases are the most common. In our case, GM occurred through hematogenous metastasis.

Current treatment methods for GM include surgical resection, neoadjuvant chemoradiotherapy, endoscopic electrocoagulation, and endoscopic resection.^[18]^ Because of rapid metastatic tumor growth, the survival time of GM patients is only a few months after primary diagnosis.^[19]^ However, with appropriate treatment, patients can live longer. By combining surgery with chemotherapy and radiotherapy, the longest survival time is 7 years.^[8]^ So far, our patient recovers well, 18 months after surgery, with 13 times of chemotherapy.

This report presents a rare case of synchronous isolated GM from ascending colon carcinoma. In this case, polypoid lesion in the gastric wall has certain imaging characteristics feature of GM on CT. This case study also demonstrates that surgery combined with chemotherapy may promote prognosis in patients with synchronous isolated GM.

## Acknowledgments

We are grateful Prof Dayue Duan, who provided *guidance* for improving our paper.

## Author contributions

All the authors have contributed to the creation of this manuscript for important intellectual and read and approved the manuscript.

**Methodology:** Bin Yang.

**Writing—original draft:** Bin Yang.

**Writing—review and editing:** Bin Yang, Guangyan Si.

**Data curation:** Zhonghua Gan, Shulan Liu.
